# Overexpression of CDCA2 in Human Squamous Cell Carcinoma: Correlation with Prevention of G1 Phase Arrest and Apoptosis

**DOI:** 10.1371/journal.pone.0056381

**Published:** 2013-02-13

**Authors:** Fumihiko Uchida, Katsuhiro Uzawa, Atsushi Kasamatsu, Hiroaki Takatori, Yosuke Sakamoto, Katsunori Ogawara, Masashi Shiiba, Hiroki Bukawa, Hideki Tanzawa

**Affiliations:** 1 Department of Oral and Maxillofacial Surgery, Clinical Sciences, Graduate School of Comprehensive Human Sciences, University of Tsukuba, Tsukuba, Ibaraki, Japan; 2 Department of Clinical Molecular Biology, Graduate School of Medicine, Chiba University, Chuo-ku, Chiba, Japan; 3 Division of Dentistry and Oral-Maxillofacial Surgery, Chiba University Hospital, Chuo-ku, Chiba, Japan; 4 Department of Molecular Genetics, Graduate School of Medicine, Chiba University, Chuo-ku, Chiba, Japan; 5 Department of Oral and Maxillofacial Surgery, Faculty of Medicine, University of Tsukuba, Tsukuba, Ibaraki, Japan; Deutsches Krebsforschungszentrum, Germany

## Abstract

Cell division cycle associated 2 (CDCA2) recruits protein phosphatase 1 to chromatin to antagonize activation of ataxia telangiectasia mutated (ATM)-dependent signal transduction. ATM kinase plays a critical role in the DNA damage response and its phosphorylation cascade to inhibit the p53-MDM2 interaction, which releases p53 to induce p21 and G1 cell-cycle arrest. However, the relevance of CDCA2 to human malignancy including oral squamous cell carcinoma (OSCC) is unknown. In the current study, we found that CDCA2 expression was up-regulated in OSCC cell lines. Functional studies with shRNA system showed that knockdown of CDCA2 significantly (*P*<0.05) inhibited cellular proliferation compared with the control cells by arresting cell-cycle progression at the G1 phase and up-regulating the cyclin-dependent kinase inhibitors (p21^Cip1^, p27^Kip1^, p15^INK4B^, and p16^INK4A^). CDCA2 knockdown also promoted apoptosis after treatment with the DNA damage reagent, cisplatin. In clinical samples, the CDCA2 protein expression level in primary OSCCs was significantly (*P*<0.05) greater than in matched normal oral tissues (67/85, 79%). Furthermore, CDCA2-positive cases were correlated significantly (*P*<0.05) with high cancer progression. Our results showed for the first time that CDCA2 frequently is overexpressed in OSCCs and might be associated closely with OSCC progression by preventing cell-cycle arrest and apoptosis.

## Introduction

The main carcinogenic agents associated with tumoral development in the upper aerodigestive tract including the oral cavity are tobacco and alcohol, which damage cells and the genetic code [1], [2]. The stability of the genome is supported by intricate machinery of repair, damage tolerance, and checkpoint pathways that counteract DNA damage [3]. A defective DNA damage response (DDR) can result in apoptosis or possibly genomic instability, unregulated cell growth, and increased cancer risk [4]. Recent reports have shown that DDR is activated in early precancerous cells as a barrier to suppress cellular proliferation and cancer progression [5]. In addition to a defective DDR, misregulated cyclin-dependent kinases (CDKs), the cell division cycle controller, are related to carcinogenesis by inducing unscheduled proliferation and genomic and chromosomal instability [6]. Understanding the functional consequences of the dysregulation of the cell-cycle apparatus and intranuclear mechanisms that signal apoptosis after DNA damage in oral squamous cell carcinoma (OSCC) will uncover novel diagnostic and therapeutic approaches.

Our microarray analysis showed that *CDCA2*, a nuclear protein that is a specific regulatory subunit for protein phosphatase 1 γ (PP1γ) [7], was one of the genes up-regulated in the OSCC-derived cells [8]. Previously, CDCA2 was found to be a member of a group of proteins whose expression was correlated with that of known cell cycle-related protein, such as CDCA1, 3, and 4–8 [9]. Several recent studies have reported that CDCA2 is a candidate factor involved in preparing mitotic chromatin for the transition to interphase [10] and controls PP1γ-dependent essential DDR regulation [11]. In addition, CDCA2 modulates phosphorylation of major mitotic histone H3 in a PP1-dependent manner [12]. Moreover, Vagnarelli *et al.* [13] reported that the CDCA2/PP1γ complex is indeed an anaphase-activated protein phosphatase regulated via CDCA2 phosphorylation. Although, the *CDCA2* gene is overexpressed in aggressive neuroblastoma tumors and melanoma cell lines [14], [15], the expression status and function of CDCA2 in OSCCs are not fully characterized.

The purpose of the current study was to investigate the potential oncogenic activities of CDCA2 and its expression profile in OSCCs. We showed the functional and clinical results of a comprehensive analysis for aberrant expression of CDCA2 in OSCCs.

## Materials and Methods

### Ethics Statement

The study protocol was approved by the Ethical Committee of Graduate School of Medicine, Chiba University (The approval number, 236) and was performed in accordance with the ethical standards laid down in the Declaration of Helsinki. Written informed consent was received from all patients.

### Cell culture

The human OSCC cell lines (HSC-2, HSC-3, HSC-4, Ca9-22, Ho-1-u-1, and Sa3) were purchased from the RIKEN BioResource Center through the National Bio-Resource Project of the Ministry of Education, Culture, Sports, Science and Technology, Tsukuba, Japan. All cell lines were HPV negative. Primary cultured human normal oral keratinocytes (HNOKs) were used as a normal control [16], [17]. The OSCC cell lines and HeLa cell line were grown in Dulbecco's modified Eagle medium (DMEM) F-12 HAM (Sigma Aldrich, St. Louis, MO) supplemented with 10% fetal bovine serum (FBS) (Sigma) and 50 units/ml penicillin and streptomycin (Sigma). HNOKs were grown in Oral Keratinocyte Medium (ScienCell Research Laboratories, Carlsbad, CA) comprised of 5 ml of oral keratinocyte growth supplement (ScienCell Research Laboratories) and 5 ml of penicillin/streptomycin solution (ScienCell Research Laboratories).

### Tissue specimens

Primary OSCC samples and corresponding normal oral tissues were obtained at the time of surgeries performed at Chiba University Hospital. All patients provided informed consent to undergo the study protocol, which was approved by the institutional review board of Chiba University. The tissues were divided into two parts, one of which was frozen immediately and stored at −80°C until RNA isolation, and the second was fixed in 20% buffered formaldehyde solution for pathologic diagnosis and immunohistochemistry (IHC). The Department of Pathology, Chiba University Hospital, performed the histopathologic diagnosis of each tissue specimen according to the World Health Organization criteria. Clinicopathological staging was determined according to the tumor-node-metastases classification of the International Union against Cancer. All OSCC samples were confirmed histologically and checked to ensure the presence of tumor in greater than 90% of specimens.

### Preparation of cDNA

Total RNA was isolated using Trizol Reagent (Invitrogen, Carlsbad, CA), according to the manufacturer's instructions. cDNA was generated from 5 µg of total RNA using Ready-To-Go You-Prime First-Strand Beads (GE Healthcare, Buckinghamshire, UK) and oligo (dT) primer (Sigma Genosys, Ishikari, Japan), according to the manufacturers' instructions.

### mRNA expression analysis

Real-time quantitative reverse transcriptase-polymerase chain reaction (qRT-PCR) was performed to evaluate the expression levels of *CDCA2* mRNA in OSCC-derived cell lines, the HeLa cell line, and the HNOKs. qRT-PCR was performed using the LightCycler 480 apparatus (Roche Diagnostics GmbH, Mannheim, Germany). Primers were designed using the ProbeFinder qPCR assay design software (Roche), which is freely accessible at www.universalprobelibrary.com. The sequences of the gene-specific primers were as follows: *CDCA2* forward 5′-ATGACCGGCTGTCTGGAAT-3′ and reverse 5′-GCTGAGACCTTCCTTTCTGGT-3′. The PCR reactions were carried out in a final volume of 20 µl of a reaction mixture comprised of 10 µl of LightCycler 480 Probes Master, 0.2 µl of universal probe (Roche), and 0.2 µM of the primers, according to the manufacturer's instructions. The reaction mixture was loaded onto the PCR plate and subjected to an initial denaturation at 95°C for 10 min, followed by 45 rounds of amplification at 95°C (10 sec) for denaturation, 60°C (30 sec) for annealing, and 72°C (1 sec) for extension, followed by a cooling step at 50°C for 30 seconds. The transcript amounts for the *CDCA2* gene was estimated from the respective standard curves and normalized to the *glyceraldehyde-3-phosphate dehydrogenase* (*GAPDH*) forward 5′-AGCCACATCGCTCAGACAC-3′ and reverse 5′-GCCCAATACGACCAAATCC-3′ transcript amounts determined in corresponding samples.

### Protein expression analysis

The cells were washed twice with cold phosphate buffered saline (PBS) and centrifuged briefly. The cell pellets were incubated at 4°C for 30 min in a lysis buffer (7 M urea, 2 M thiourea, 4% w/v CHAPS, and 10 mM Tris pH 7.4) with a proteinase inhibitor cocktail (Roche). The protein concentration was measured using the Bradford reagent (Bio-Rad, Richmond, CA). Protein extracts were electrophoresed on 4% to 12% Bis-Tris gel, transferred to nitrocellulose membranes (Invitrogen), and blocked for 1 hr at room temperature with Blocking One (Nacalai Tesque, Inc., Kyoto, Japan). The membranes were washed three times with 0.1% Tween-20 in Tris-buffered saline and incubated with antibody for CDCA2 (Sigma), p21^Cip1^, p27^Kip1^, p15^INK4B^, p16^INK4A^, CDK4, CDK6, Cyclin D1 (Cell Signaling Technology, Danvers, MA), Cyclin E, phosphorylated-ATM (p-ATM) (Ser1981), p53, phosphorylated-p53 (p-p53) (Ser 15), p-p53 (Ser 46), poly (ADP-ribose) polymerase 1 (PARP-1) (full-length PARP-1 [116 kDa]), and large-fragment PARP-1 (85 kDa) (Santa Cruz Biotechnology, Santa Cruz, CA) overnight at 4°C and α-tubulin (Santa Cruz Biotechnology) for 1 hr at room temperature. The membranes were washed again and incubated with anti-rabbit or anti-mouse IgG (H+L) horseradish peroxidase conjugate (Promega, Madison, WI) as a secondary antibody for 1 hr at room temperature. Finally, the membranes were detected using SuperSignal West Pico Chemiluminescent substrate (Thermo, Rockford, IL), and immunoblotting was visualized by exposing the membranes to ATTO Light-Capture II (Tokyo, Japan). Signal intensities were quantitated using the CS Analyzer version 3.0 software (ATTO).

### Stable transfection of CDCA2 shRNA

Stable transfection was performed at about 80% confluency in 24-well plates using Lipofectamine LTX and Plus Reagents (Invitrogen), according to the manufacturer's instructions. Briefly, a total of 2×10^5^ cells (Sa3 and Ca9-22) were seeded into each well in DMEM F-12 HAM containing 10% FBS without antibiotics. CDCA2 shRNA (shCDCA2) and the control shRNA (0.1 µg) (mock) (Santa Cruz Biotechnology) vectors were transfected with 0.5 µl of Plus Reagents and 1.25 µl of Lipofectamine LTX. After transfection, the cells were isolated by the culture medium containing 2 µg/mL puromycin (Invitrogen). After 3 to 4 weeks, resistant cell clones were picked and transferred to 6-well plates and gradually expanded to 10-cm dishes. At 90% confluence, qRT-PCR and Western blot analyses were performed to assess the efficiency of CDCA2 knockdown.

### Cellular growth

To evaluate the effect of CDCA2 knockdown on cellular proliferation, we analyzed cellular growth in shCDCA2- and mock-transfected cells. These transfectants were seeded in 6-well plates at a density of 1×10^4^ viable cells per well. The experiments were carried out for 168 hr, and the cells were counted every 24 hr. At the indicated time point, the cells were trypsinized and counted using a hemocytometer in triplicate samples.

### Cell-cycle analysis

To assess cell-cycle distribution of the entire cell populations, the cells were harvested at the time of 168 hr after cell seeding, washed with PBS, and probed with Cycletest Plus DNA reagent kit (Becton-Dickinson, San Jose, CA), according to the manufacturer's protocol. Briefly, the cells were centrifuged at 400×g for 5 min. The cell pellets were resuspended with 250 µl of trypsin buffer, and incubated for 10 min at room temperature. We then added 200 µl of trypsin inhibitor and RNase buffer. Finally, the cells were labeled with 200 µl of propidium iodide stain solution. Flow cytometric determination of the DNA content was analyzed by FACScalibur (Becton-Dickinson). The fractions of the cells in the G0-G1, S, and G2-M phases were analyzed using Flow Jo software (Tree Star, Ashland, OR).

### MTT [3-(4,5-dimethyl-thiazol-2-yl)-2,5-diphenyltetrazolium bromide] assay

To assess chemosensitivity to cisplatin (CDDP) (Sigma), clinically used DNA-damaging agents, we determined the proliferation rates using the MTT assay (Funakoshi, Tokyo, Japan) via a protocol described previously [18]. Briefly, the cells were seeded in 96-well plates at 2×10^3^ cells/well with DMEM F-12 HAM containing 10% FBS with CDDP for 72 hr. Thereafter, the number of cells was quantified with the MTT cell growth assay kit. Six wells were used for each concentration, and the experiment was repeated 3 times. The 50% inhibitory concentration (IC50) was calculated from the survival curve.

### Apoptosis assay

Apoptosis detection was carried out using a terminal deoxynucleotidyl transferase-mediated deoxyuridine triphosphate-biotin nick end labeling (TUNEL) method with an In situ Apoptosis Detection Kit (Takara, Shiga, Japan), according to the manufacturer's protocol. Briefly, the cells were treated with 1 µM of CDDP for 72 hr and fixed in 4% neutral buffered formalin, dried onto microscope slides, washed with PBS, equilibrated, and incubated with terminal deoxynucleotidyl transferase in a reaction buffer at 37°C for 1 hr. The specimens were washed with PBS to stop the reaction, and the slides were incubated with anti-digoxigenin antibody coupled with Fluorescein isothiocyanate for 30 min at room temperature. The specimens were washed three times with PBS before mounting for photomicrography under phase and epifluorescence illumination. The apoptotic index was determined by calculating the percentage of cells that was apoptotic through positive staining. All slides were blindly evaluated three times.

### IHC

IHC of 4-µm sections of paraffin-embedded specimens was performed using rabbit anti-CDCA2 polyclonal antibody and rabbit anti-Ki-67 polyclonal antibody (Santa Cruz Biotechnology). Briefly, after deparaffinization and hydration, the endogenous peroxidase activity was quenched by 30-min incubation in a mixture of 0.3% hydrogen peroxide solution in 100% methanol, after which the sections were blocked for 2 hr at room temperature with 1.5% blocking serum (Santa Cruz Biotechnology) in PBS before reaction overnight with antibody for CDCA2 (1∶100 dilution) and Ki-67 (1∶50 dilution) at 4°C in a moist chamber. Upon incubation with the primary antibody, the specimens were washed three times in PBS and treated with Envision reagent (DAKO, Carpinteria, CA) followed by color development in 3,3′-diaminobenzidine tetrahydrochloride (DAKO). The slides then were lightly counterstained with hematoxylin, dehydrated with ethanol, cleaned with xylene, and mounted. Non-specific binding of an antibody to proteins other than the antigen sometimes occurred. To avoid non-specific binding, an immunizing peptide blocking experiment was performed. As a negative control, triplicate sections were immunostained without exposure to primary antibodies, which confirmed the staining specificity. To quantify the status of the CDCA2 protein expression in those components, we used an IHC scoring systems described previously [19]–[23]. This IHC scoring system was established to quantitatively evaluate the IHC staining. The stained cells were determined in at least five random fields at 400× magnification in each section. We counted 300 cells per one field of vision. The staining intensity (1, weak; 2, moderate; 3, intense) and the number of positive cells in the field of vision were then multiplied to calculate the IHC score using the following formula: IHC score = 1×(number of weakly stained cells in the field)+2×(number of moderately stained cells in the field)+3×(number of intensely stained cells in the field). Cases with a CDCA2 IHC score exceeding 90.0 (the maximal score within+3 standard deviations [SD] of the mean of normal tissues) were defined as CDCA2-positive, because 100% of the distribution falls within ±3 SD of the mean in normal tissues. Two independent pathologists masked to the patients' clinical status made these judgments. We also analyzed the number of cells stained positive for Ki-67 on 46 OSCCs that were extracted at random from the CDCA2 negative group (23 cases) and CDCA2 positive group (23 cases) as a measure of the proliferation. Results were expressed as the number of Ki-67-positive cells/field counted (five random fields per slide, magnification ×100).

### Statistical analysis

Statistical significance was determined using Fisher's exact test or the Mann-Whitney's *U* test. *P*<0.05 was considered significant. The data are expressed as the mean ± standard error of the mean (SEM).

## Results

### Overexpression of CDCA2 in OSCC-derived cell lines and the HeLa cell line

**Figure 1:**
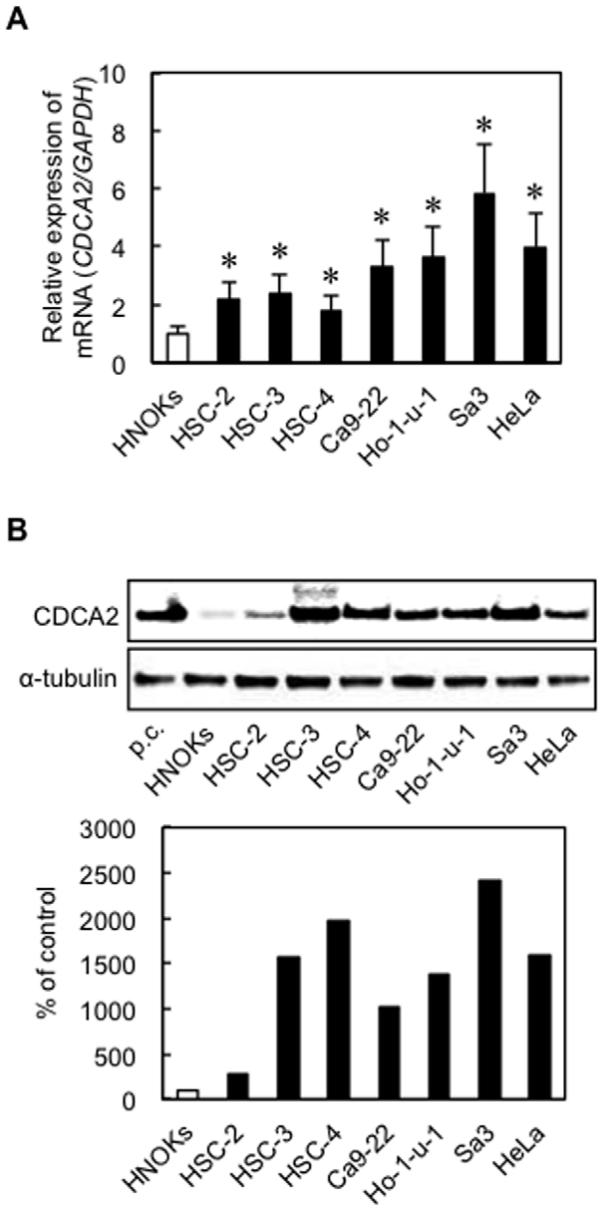
Evaluation of CDCA2 expression in OSCC-derived cell lines and the HeLa cell line. (**A**) Quantification of *CDCA2* mRNA expression in OSCC-derived cell lines and the HeLa cell line by qRT-PCR analysis. Significant up-regulation of *CDCA2* mRNA is seen in all cell lines compared with that in HNOKs (*P<0.05, Mann-Whitney's *U* test). Data are expressed as the means ± SEM of triplicate results. (**B**) Western blot analysis of CDCA2 in the OSCC cell lines, the HeLa cell line, and the HNOKs. CDCA2 protein expression is up-regulated in all cell lines compared with HNOKs. Densitometric CDCA2 protein data are normalized to α-tubulin protein levels. The values are expressed as a percentage of the HNOKs. p.c., positive control (HepG2 (Human hepatocellular liver carcinoma cell line) Nuclear Lysate).

To investigate mRNA and protein expression of CDCA2 identified as a cancer-related gene in our previous microarray data [8], we performed qRT-PCR and Western blot analyses using six OSCC-derived cell lines (HSC-2, HSC-3, HSC-4, Ca9-22, Ho-1-u-1, and Sa3), the HeLa cell line, and the HNOKs. *CDCA2* mRNA was significantly (*P<0.05) up-regulated in all OSCC-derived cell lines and the HeLa cell line compared with the HNOKs (Figure 1A). Figure 1B shows representative results of Western blot analysis. The molecular weight of CDCA2 was 112 kDa. All cell lines had a significant increase in CDCA2 protein expression compared with the HNOKs. Expression analysis indicated that both transcription and translation products of this molecule were highly expressed in OSCC-derived cell lines and the HeLa cell line.

### Establishment of CDCA2 knockdown cells

**Figure 2:**
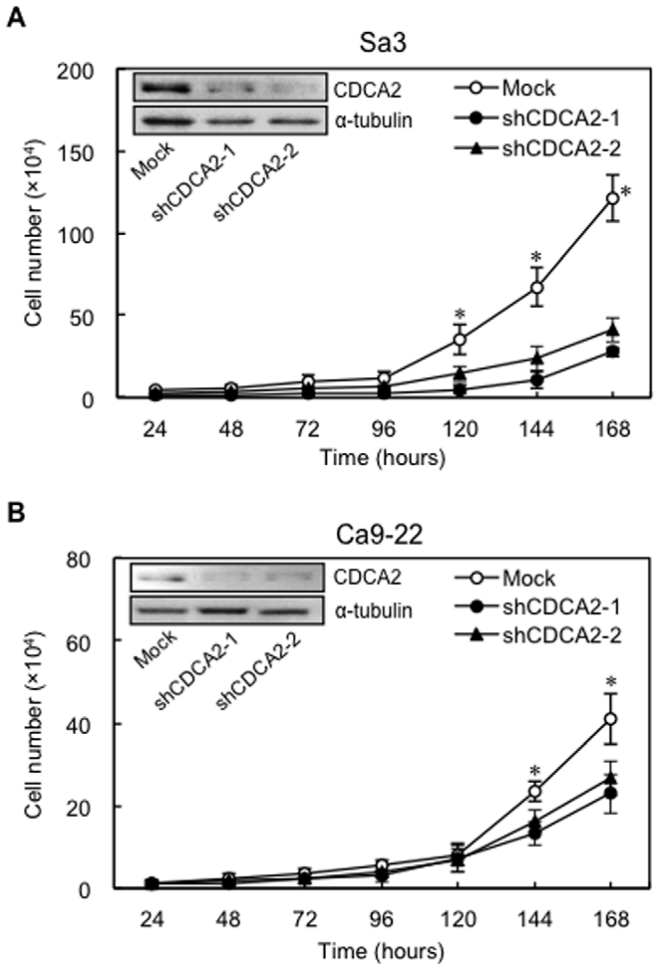
Proliferation of shCDCA2-transfected Sa3 and Ca9-22 cells. The shCDCA2-transfected Sa3 (**A**) and Ca9-22 (**B**) cells (shCDCA2-1 and shCDCA2-2 correspond to two selected clones) show a significant (*P<0.05, Mann-Whitney's *U* test) decrease in cellular growth compared with the mock-transfected cells. The results are expressed as the means ± SEM of values from three assays. Representative Western blot data show that CDCA2 proteins are markedly down-regulated in shCDCA2-transfected Sa3 (**A**) and Ca9-22 (**B**) cells. Densitometric CDCA2 protein data are shown in Figure S1C and D.

Sa3 and Ca9-22 cells were transfected with shCDCA2 and mock plasmids. qRT-PCR and Western blot analyses were performed to assess the efficiency of CDCA2 knockdown. *CDCA2* mRNA expression in shCDCA2-transfected cells was significantly (*P<0.05) lower than that in mock-transfected cells (Figure S1A and B). The CDCA2 protein levels in the shCDCA2-transfected Sa3 (Figure 2A) and Ca9-22 (Figure 2B) cells also decreased markedly compared with the mock-transfected cells. The densitometric CDCA2 protein levels in shCDCA2-transfected cells decreased markedly compared with the levels in the mock-transfected cells (Figure S1C and D).

### Reduced cellular growth in CDCA2 knockdown cells

To investigate the antiproliferative effects in shCDCA2-transfected cells, cellular growth was monitored for 168 hr. The shCDCA2-transfected Sa3 (Figure 2A) and Ca9-22 (Figure 2B) cells showed a significant decrease in cellular growth compared with mock-transfected cells (*P<0.05).

### Knockdown of CDCA2 promotes cell-cycle arrest

**Figure 3:**
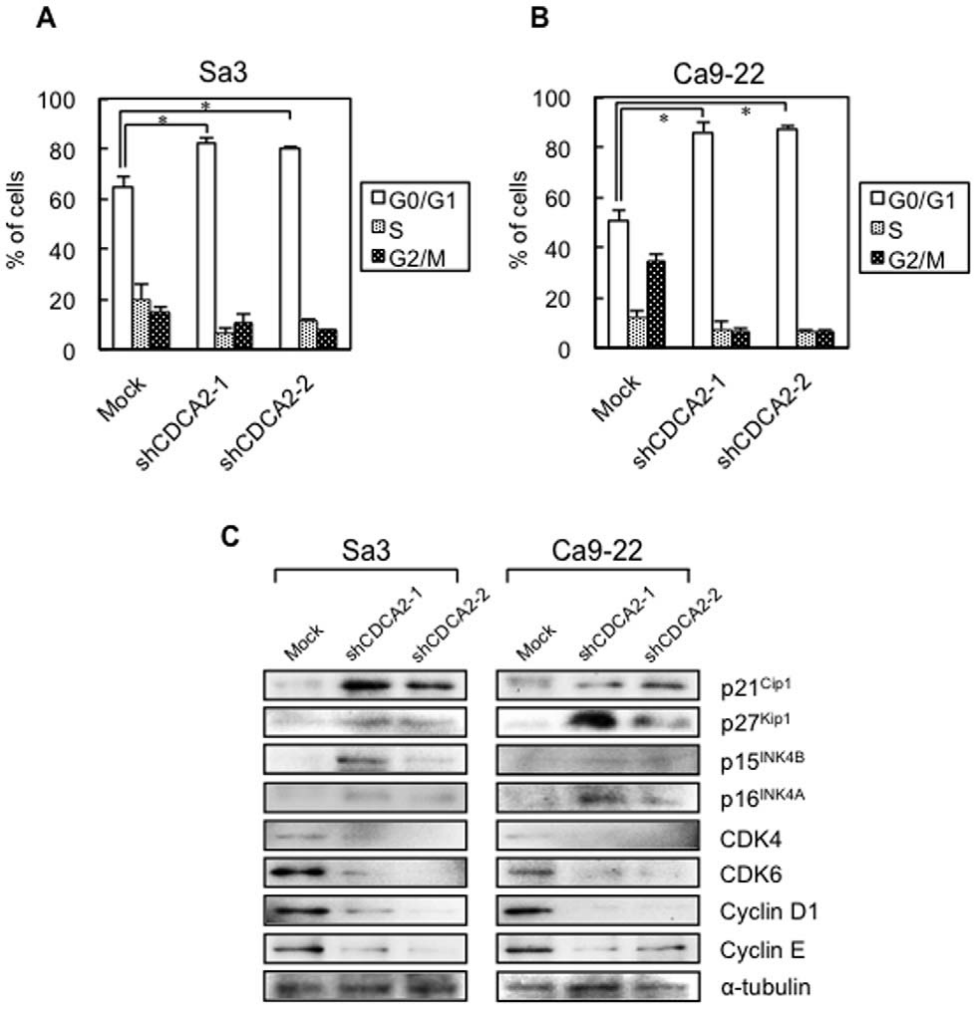
Flow cytometric determination of DNA content and expression of cell-cycle regulators in shCDCA2-transfected cells. We analyzed the flow cytometric determination of DNA content by a FACScalibur in the G0-G1, S, and G2-M phases. We then determined the protein expression level of the CDKIs (p21^Cip1^, p27^Kip1^, p15^INK4B^, and p16^INK4A^), CDK4, CDK6, Cyclin D1, and Cyclin E to identify the mechanism by which CDCA2 blocks G1 progression. (**A**) Representative FACS analysis shows that the number of cells in the G0/G1 phase is significantly (*P<0.05, Mann-Whitney's *U* test) increased in shCDCA2-transfected Sa3 and Ca9-22 cells. (**B**) Western blot analysis shows the protein expressions of CDKIs, CDKs, and Cyclins. The protein expression data show up-regulation of p21^Cip1^, p27^Kip1^, p15^INK4B^, and p16^INK4A^ and down-regulation of CDK4, CDK6, Cyclin D1, and Cyclin E in the CDCA2 knockdown cells. Densitometric protein data are shown in Figure S2A and B.

To investigate the mechanism by which down-regulated CDCA2 is related to cell-cycle progression, we performed fluorescence-activated cell sorting (FACS) analysis of the shCDCA2-transfected cells. A representative FACS analysis of shCDCA2- and mock-transfected cells is shown in Figure 3A and B. The percentage of the G0/G1 phase in shCDCA2-transfected cells was significantly (*P<0.05) higher than in mock-transfected cells. To identify the mechanism by which down-regulated CDCA2 blocks G1 progression, we assessed the protein expression level of cyclin-dependent kinase inhibitors (CDKIs) (p21^Cip1^, p27^Kip1^, p15 ^INK4B^, p16^INK4A^), CDK4, CDK6, Cyclin D1, and Cyclin E (Figure 3C, Figure S2A and B). The protein expression data showed up-regulation of CDKIs (p21^Cip1^, p27^Kip1^, p15^INK4B^, and p16^INK4A^) and down-regulation of CDK4, CDK6, Cyclin D1, and Cyclin E in the CDCA2 knockdown cells.

### Knockdown of CDCA2 induces apoptosis by activating the DDR

**Figure 4:**
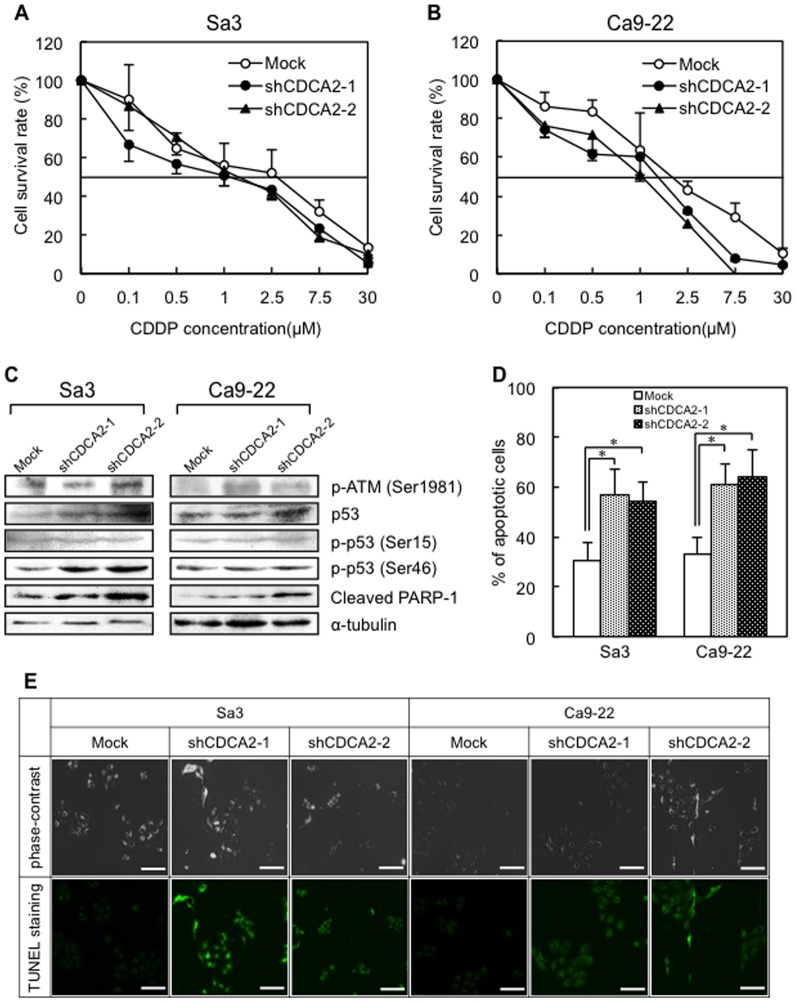
Antitumor activity in shCDCA2-transfected cells. Sensitivity of shCDCA2-transfected Sa3 (**A**) and Ca9-22 (**B**) cells to CDDP. The IC50 values for the shCDCA2-transfected cells were Sa3 (Mock: 2.78 µM, shCDCA2-1: 1.12 µM, shCDCA2-2: 1.31 µM) and Ca9-22 (Mock: 1.82 µM, shCDCA2-1: 1.39 µM, shCDCA2-2: 1.04 µM), respectively. (**C**) Western blot analysis of p-ATM, p53, p-p53 (Ser15), p-p53 (Ser46), and cleaved PARP-1 in the shCDCA2- and mock-transfected cells after CDDP treatment. p -ATM, p-p53 (Ser46), and cleaved PARP-1 protein expression is up-regulated in the shCDCA2-transfected cells compared with the mock-transfected cells; the p53 and p-p53 (Ser15) level is unchanged. Densitometric protein data are shown in Figure S3A and B. (**D**) Quantitative analysis using the TUNEL assay. The number of dead cells per field of view after CDDP treatment is significantly increased in the shCDCA2-transfected cells compared with the mock-transfected cells (*P<0.05, Mann-Whitney's *U* test). (**E**) Representative results of the TUNEL assay (Scale bars, 30 µm.). More apoptotic cells are clearly seen in the shCDCA2 transfected cells than in the mock transfected cells.

The expression level of CDCA2 protein determines the activation of the DNA checkpoint [11]. We therefore assessed the effect of CDDP treatment for sensitivity to DNA damage in CDCA2 knockdown cells. The IC50 values for CDDP in the mock-transfected cells were 2.1 to 2.5-fold and 1.3 to 1.8-fold that in the shCDCA2-transfected Sa3 (Figure 4A) and Ca9-22 (Figure 4B). The shCDCA2-transfected cells were more sensitive to CDDP compared with mock-transfected cells. We then assessed whether the cleaved PARP-1, the protein marker for cell apoptosis and its cleavage reflects apoptotic cell death [24], was detectable in shCDCA2-transfected cells after treated with 1 µM of CDDP for 72 hr. Cleaved PARP-1 in the shCDCA2-transfected cells increased compared to the mock-transfected cells (Figure 4C, Figure S3A and B). To investigate a potential underlying mechanism that would explain induced sensitivity for CDDP in the shCDCA2-transfected cells, we assessed the ATM-dependent signaling pathways. With 1 µM of CDDP treatment for 72 hr, p-ATM and p-p53 (Ser46) protein increased in shCDCA2-transfected cells compared with mock-transfected cells (Figure 4C, Figure S3A and B). Further, the number of TUNEL-positive cells in the shCDCA2-transfected cells also significantly (*P<0.05) increased compared to the mock-transfected cells (Figure 4D). Figure 4E shows representative results of the TUNEL assay, which indicated that CDCA2 knockdown induced apoptotic cell death.

### Expression of CDCA2 and Ki-67 and clinicopathological variables of primary OSCCs

**Figure 5:**
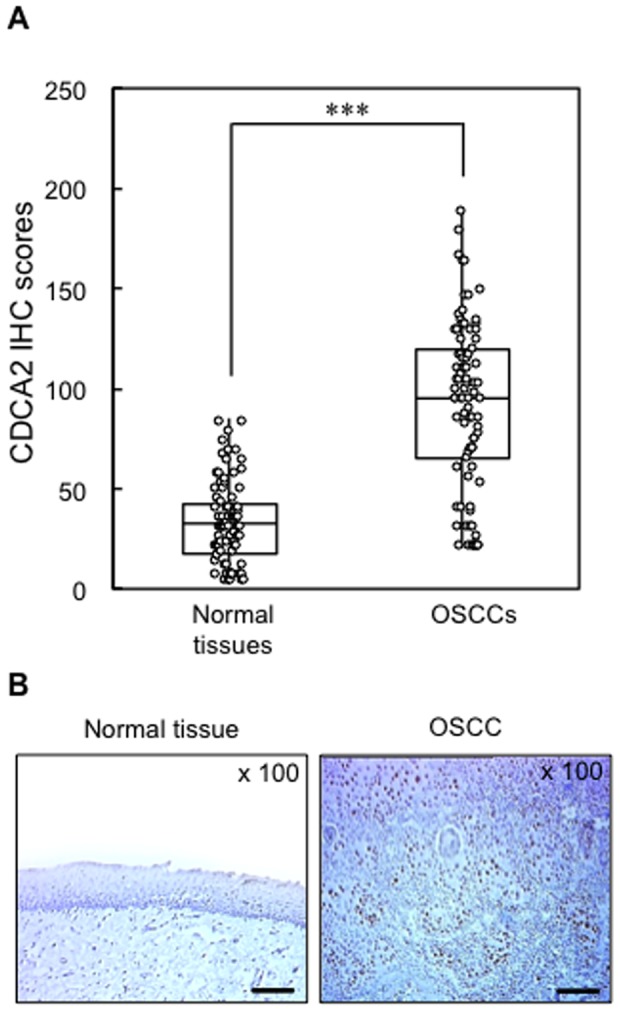
Evaluation of CDCA2 expression in normal oral tissues and primary OSCCs. (**A**) The status of CDCA2 protein expression in primary OSCCs and paired normal oral tissues from 85 patients based on the IHC scoring system. The CDCA2 IHC scores of normal oral tissues and OSCCs range from 2.5 to 90.0 (median, 30.0) and 20.0 to 190.0 (median, 95.0), respectively. The CDCA2 protein expression level in OSCCs is significantly higher (***P<0.001, Mann-Whitney's *U* test) than that in normal oral tissues. (**B**) Representative IHC results of CDCA2 in normal oral tissue and primary OSCC (×100 magnification. Scale bars, 100 µm). Strong CDCA2 immunoreaction is detected in OSCCs, whereas the normal oral tissues show almost negative immunostaining.

We analyzed the CDCA2 protein expression in primary OSCCs and paired normal oral tissues from 85 patients using the IHC scoring system. The CDCA2 IHC scores of the primary OSCCs and normal oral tissues ranged from 20.0 to 190.0 (median, 95.0), and 2.5 to 90.0 (median, 30.0), respectively. The IHC scores in primary OSCCs were significantly (***P<0.001) higher than those in normal oral tissues (Figure 5A). Figure 5B shows representative IHC results for CDCA2 protein in normal oral tissues and primary OSCCs. Strong CDCA2 immunoreactivity was detected in the nucleus in the OSCCs, whereas normal oral tissues showed weak immunostaining. Similar to the data from the OSCC-derived cell lines, CDCA2 protein expression was up-regulated in 67 (79%) of 85 primary OSCCs compared with the matched normal oral tissues. Table 1 shows the correlations between the clinicopathologic characteristics of the patients with OSCC and the status of the CDCA2 protein expression using the IHC scoring system. Among the clinical classifications, CDCA2-positive OSCCs were correlated significantly (*P<0.05) with tumor size and TNM staging of OSCC. The number of cells stained positive for Ki-67 in OSCCs significantly (***P<0.001) reduced in CDCA2 negative group in comparison to CDCA2 positive group (Figure S4).

**Table 1:** Correlation between CDCA2 expression and clinical classification in OSCCs.

		Results of immunostaining		
Clinical classification		No. patients (%)		
	Total	CDCA2- negative	CDCA2- positive	*P* value
Age at surgery (years)				
<60	22	8 (36)	14 (64)	0.637
≧60, <70	22	10 (45)	12 (55)	
≧70	41	18 (44)	23 (56)	
Gender				
Male	52	22 (42)	30 (58)	0.775
Female	33	15 (45)	18 (55)	
T-primary tumor				
T1	16	12 (75)	4 (25)	0.041*
T2	34	12 (35)	22 (65)	
T3	19	6 (32)	13 (68)	
T4	16	6 (38)	10 (62)	
T1+T2	50	24 (48)	26 (52)	0.210
T3+T4	35	12 (34)	23 (66)	
N-regional lymph node				
N (−)	43	16 (37)	27 (63)	0.234
N (+)	42	21 (50)	21 (50)	
Stage				
I	15	12 (80)	3 (20)	0.049*
II	27	7 (26)	20 (74)	
III	19	7 (37)	12 (63)	
IV	24	8 (33)	16 (67)	
Histopathologic type				
Well differentiated	46	17 (37)	29 (63)	0.302
Moderately differentiated	34	14 (41)	20 (59)	
Poorly differentiated	5	4 (80)	1 (20)	
Tumor site				
Gingiva	29	15 (52)	14 (48)	0.574
Tongue	45	16 (36)	29 (64)	
Buccal mucosa	7	3 (43)	4 (57)	
Oral floor	4	2 (50)	2 (50)	

* *P*<0.05.

## Discussion

The current study provided the first evidence that CDCA2 overexpression occurs widely in OSCCs and is positively correlates with tumoral progression and advanced disease stages. Consistent with these clinical findings, experiments on oral cancer cell lines showed that suppression of CDCA2 expression with shRNA significantly inhibits cellular proliferation by arresting cell-cycle progression at the G1 phase and inducing apoptotic cell death by treatment with CDDP through activation of the DDR of these OSCC cells *in vitro*. Therefore, these results suggest that CDCA2 may play a significant role in the cellular proliferation and/or the apoptotic response in human OSCCs.

Little is known about the mechanism underlying the aberrant expression of CDCA2 in malignant cells. In addition to previous studies that reported CDCA2 up-regulation in aggressive neuroblastoma, melanoma, and breast cancer [11], [14], [15], we found significant up-regulation of CDCA2 in the OSCC cell lines compared with that in the HNOKs. Since Trinkle-Mulcahy *et al.* [7] reported the specific characterization and functions of CDCA2 in the HeLa cells and showed that recruiting of PP1γ to chromatin was essential for cellular viability, we used the cell as the positive control in the current study (data not shown). Our results identified CDCA2 expression in human OSCCs and therefore parallel findings that CDCA2 is overexpressed in malignant tumor cell lines [11], [14], [15].

Peng *et al.* [11] also reported that CDCA2 recruits PP1γ to chromatin to antagonize activation of ATM-dependent signal transduction and that CDCA2-dependent DDR regulation is strengthened by CDCA2 overexpression during cancer progression, resulting in reduced DDR sensitivity. After DNA damage, cell-cycle checkpoints are activated. DNA damage-inducible cell-cycle checkpoints transiently delay cell-cycle progression in proliferating cells, presumably providing time for repair [25], [26]. DNA damage checkpoint control arises at multiple points in the cell cycle including late G1, intra-S phase, and the G2 phase [27]. Checkpoint activation is controlled by two master kinases, ATM and ataxia-telangiectasia mutated related (ATR) [28]. ATM responds to DNA double-strand breaks and disruptions in chromatin structure and activated ATM phosphorylates p53 at Ser 15, which inhibits the binding of MDM2 to p53 [29]. In cells with wild-type p53, this stabilized p53 induces transcription of the CDK inhibitor p21^Cip1^, but not apoptotic target genes, preventing CDK4 and/or CDK6 and CDK2-mediated G1/S transition [30]–[33]. Activated ATM also mediates phosphorylation of p53 at Ser 46, which is important for inducing apoptosis in response to DNA damage [34]. To determine whether the CDCA2 function is relevant to OSCC progression, we performed the shCDCA2 experiment and found that cellular proliferation decreased significantly as a result of cell-cycle arrest at the G1 phase in CDCA2 knockdown cells with up-regulation of p21^Cip1^, p27^Kip1^, p15 ^INK4B^, and p16^INK4A^. These results were consistent with the observations that cell-cycle progression is negatively controlled by CDKIs, such as p21^Cip1^, p27^Kip1^, p57^Kip2^, and the INK4 families (p15^INK4B^, p16^INK4A^, p18^INK4C^, and p19^INK4D^), which are involved in cell-cycle arrest at the G1 phase and have several functions as tumor suppressor genes [35]. CDKIs are proteins that interact with the Cyclin-CDK complex to block kinase activity, usually during G1 or in response to signals from the environment or from damaged DNA [36], [37]. We found that down-regulation of CDK4, CDK6, Cyclin D1, and Cyclin E occurred concurrently with up-regulation of CDKIs in CDCA2 knockdown cells. Our results provide initial findings that CDCA2 knockdown up-regulates expression levels of p27^Kip1^ and INK4 family genes (p15^INK4B^ and p16^INK4A^).

Furthermore, shRNA-mediated CDCA2 knockdown significantly sensitized Sa3 and Ca9-22 cells to CDDP-induced apoptosis. Our results support previous findings that CDCA2 modulates DDR sensitivity through activation of ATM-dependent signal transduction and regulates response to DNA damage by modulating expression of phosphorylated p53 at Ser 46 [34]. This suggested that CDCA2 suppression might have considerable potential in enhancing the therapeutic effects of irradiation and anticancer drugs that cause DNA damage.

We showed the status of CDCA2 expression in clinical tissue samples obtained from primary OSCCs and corresponding normal tissue by IHC staining, and this is the first study to evaluate CDCA2 expression in a large number of malignant clinical samples. We observed CDCA2 overexpression in most oral cancer specimens, and its nuclear accumulation increased with tumoral progression and advanced-tumor stage (Table 1). Moreover CDCA2 positive OSCCs were expression correlated with the expression of Ki-67. These findings indicate that CDCA2 overexpression might be linked to human oral cancer proliferation and have an important role in OSCC development and progression.

In conclusion, we found that CDCA2 is frequently overexpressed in OSCCs and plays an important role in OSCC progression by preventing the arrest of cell-cycle progression at the G1 phase via decreased expression of CDKIs and regulation of the DDR. Higher expression of CDCA2 is positively correlates with advanced-stage OSCCs. Therefore, CDCA2 would be a novel target for preventing and treating OSCC progression.

## Supporting Information

Figure S1 **mRNA and protein expression in the shCDCA2-transfected cells using qRT-PCR and Western blot analyses.** (**A, B**) *CDCA2* mRNA levels in the shCDCA2-transfected cells. qRT-PCR shows that *CDCA2* is down-regulated in the shCDCA2-transfected cells compared with the mock-transfected cells (*P<0.05, Mann-Whitney's *U* test). Data are expressed as the means ± SEM of triplicate results. (**C, D**) The densitometric CDCA2 protein levels in the shCDCA2- and the mock-transfected cells show that CDCA2 protein is markedly decreased in the shCDCA2-transfected cells compared with the mock-transfected cells.
(TIF)

Figure S2 **Quantification of protein expression in shCDCA2- and mock-transfected cells.** (**A**, **B**) The densitometric protein data are normalized to α-tubulin protein levels. The values are expressed as a percentage of the Mock. Western blot analysis shows up-regulation of p21^Cip1^, p27^Kip1^, p15^INK4B^, and p16^INK4A^ and down-regulation of CDK4, CDK6, Cyclin D1 and Cyclin E in the CDCA2 knockdown cells.
(TIF)

Figure S3 **Quantification of protein expression in shCDCA2- and mock-transfected cells after CDDP treatment.** (**A**, **B**) The densitometric protein data are normalized to α-tubulin protein levels. The values are expressed as a percentage of the Mock. Western blot analysis shows up-regulation of p-ATM, p-p53 (Ser46), and cleaved PARP-1 in the CDCA2 knockdown cells; the p53 and p-p53 (Ser15) level is unchanged.
(TIF)

Figure S4 **Evaluation of Ki-67 expression in CDCA2 negative and positive group.** The percentage of cells stained positive for Ki-67 on 46 OSCCs that were extracted at random from the CDCA2 negative group (23 cases) and CDCA2 positive group (23 cases) based on the IHC. The number of cells stained positive for Ki-67 in CDCA2 positive group significantly higher (***P<0.001, Mann-Whitney's *U* test) than that in CDCA2 negative group.
(TIF)
