# Supplementary figures and images for: Overexpression of CDCA2 in Human Squamous Cell Carcinoma: Correlation with Prevention of G1 Phase Arrest and Apoptosis
Source: PLoS One. 2013 Feb 13;8(2):e56381. doi: 10.1371/journal.pone.0056381 (PMC3572040; doi:10.1371/journal.pone.0056381)

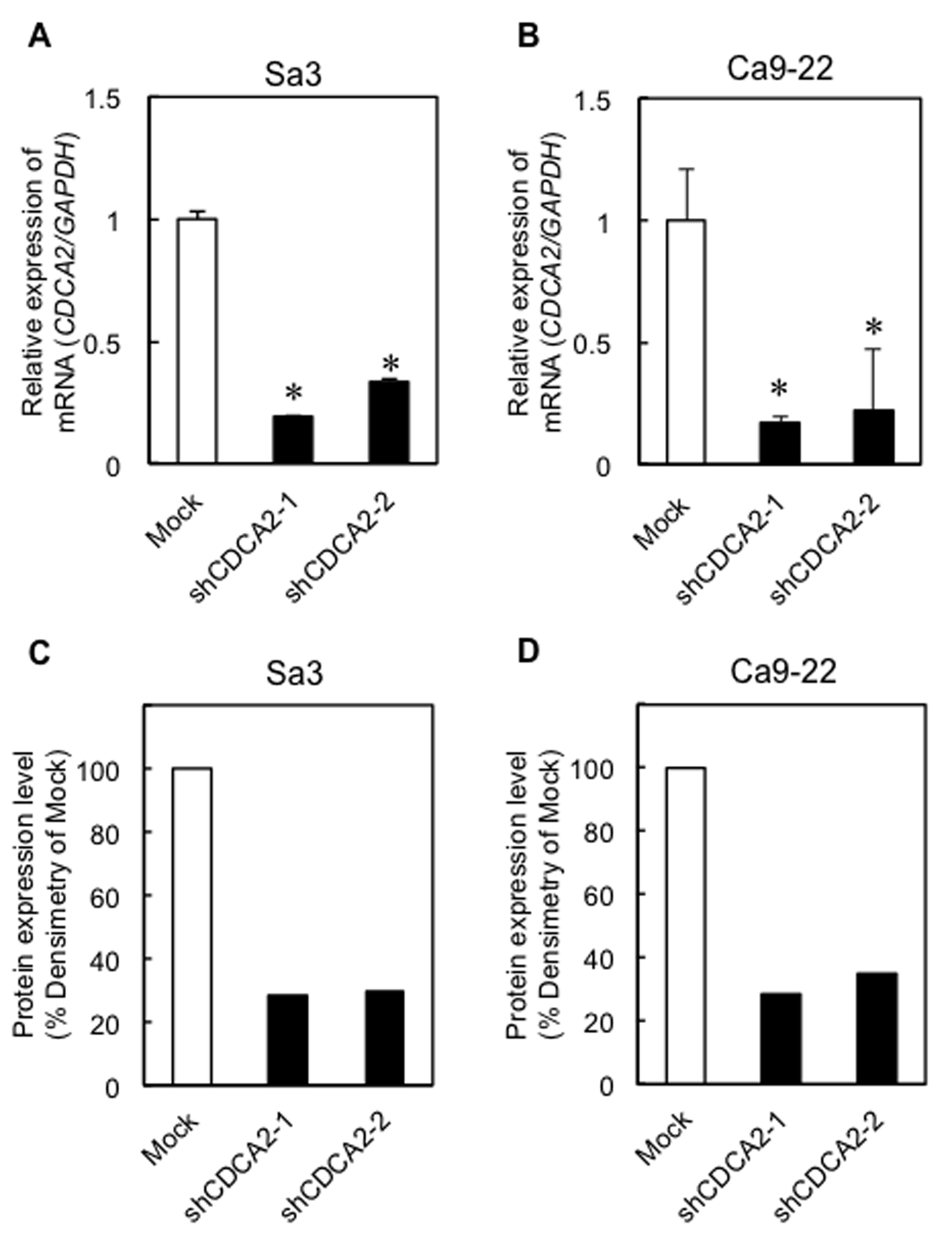

Supplement: Figure S1 — mRNA and protein expression in the shCDCA2-transfected cells using qRT-PCR and Western blot analyses. (A, B) CDCA2 mRNA levels in the shCDCA2-transfected cells. qRT-PCR shows that CDCA2 is down-regulated in the shCDCA2-transfected cells compared with the mock-transfected cells (*P<0.05, Mann-Whitney's U test). Data are expressed as the means ± SEM of triplicate results. (C, D) The densitometric CDCA2 protein levels in the shCDCA2- and the mock-transfected cells show that CDCA2 protein is markedly decreased in the shCDCA2-transfected cells compared with the mock-transfected cells. (TIF) [file pone.0056381.s001.tif]

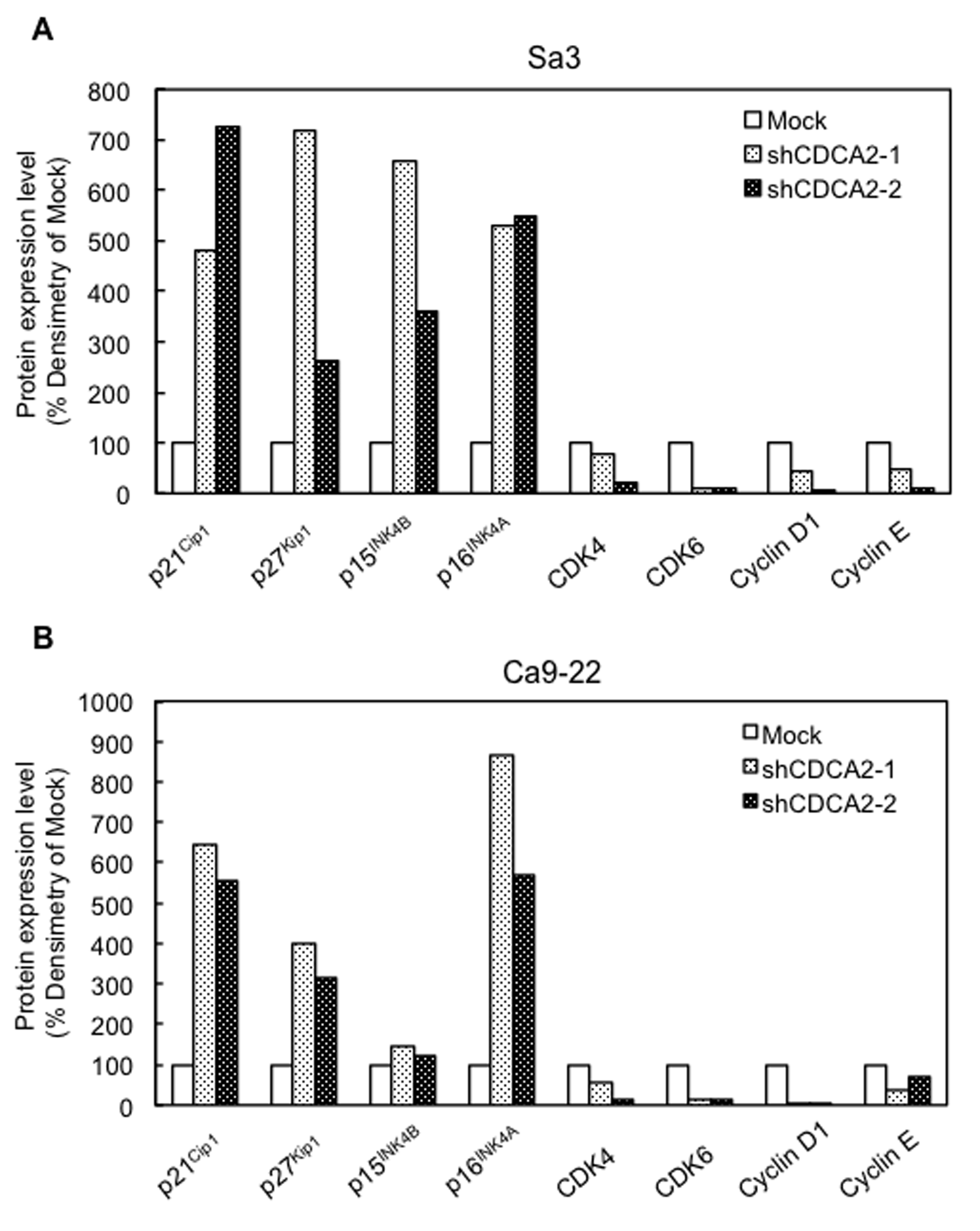

Supplement: Figure S2 — Quantification of protein expression in shCDCA2- and mock-transfected cells. (A, B) The densitometric protein data are normalized to α-tubulin protein levels. The values are expressed as a percentage of the Mock. Western blot analysis shows up-regulation of p21Cip1, p27Kip1, p15INK4B, and p16INK4A and down-regulation of CDK4, CDK6, Cyclin D1 and Cyclin E in the CDCA2 knockdown cells. (TIF) [file pone.0056381.s002.tif]

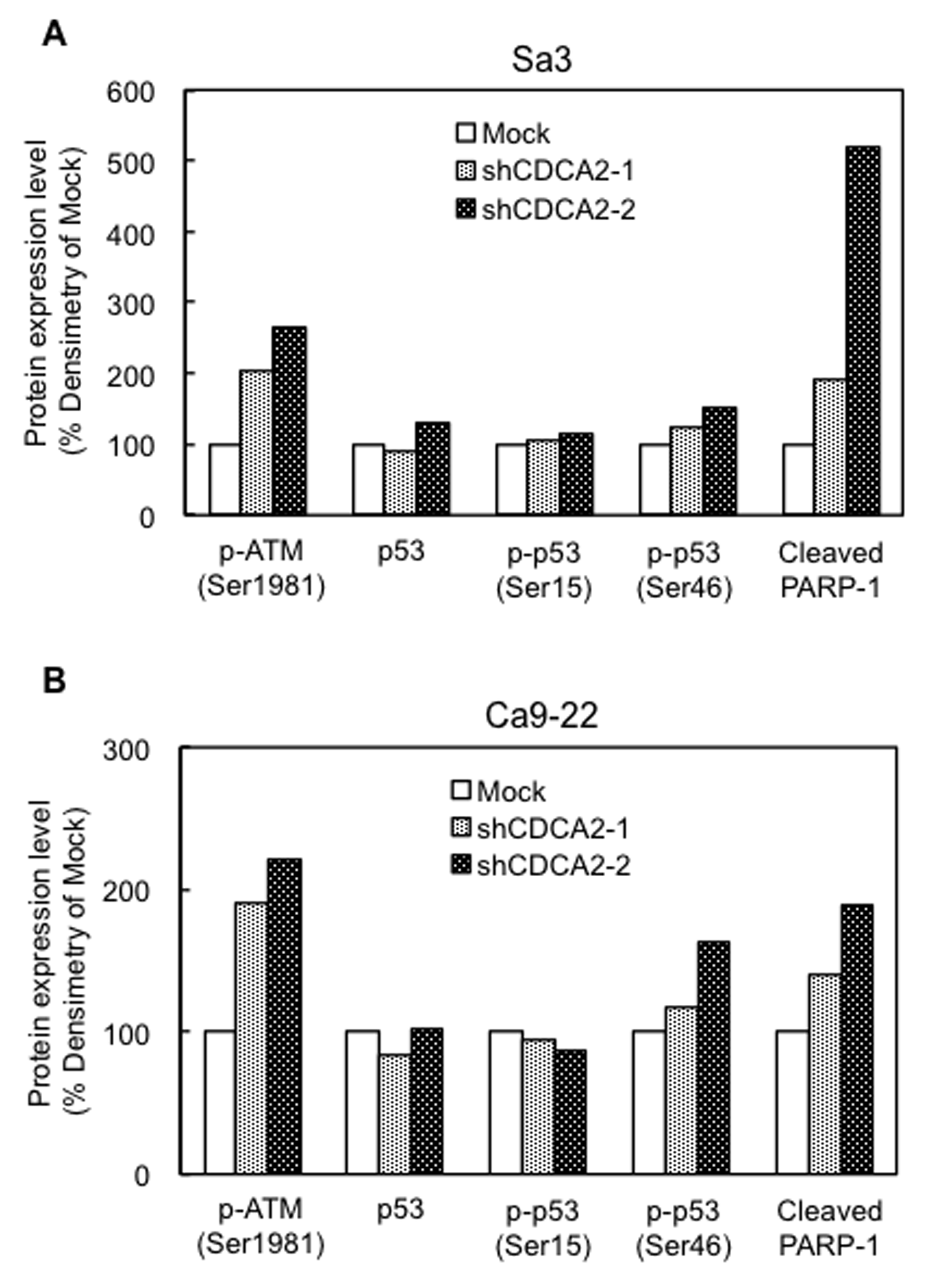

Supplement: Figure S3 — Quantification of protein expression in shCDCA2- and mock-transfected cells after CDDP treatment. (A, B) The densitometric protein data are normalized to α-tubulin protein levels. The values are expressed as a percentage of the Mock. Western blot analysis shows up-regulation of p-ATM, p-p53 (Ser46), and cleaved PARP-1 in the CDCA2 knockdown cells; the p53 and p-p53 (Ser15) level is unchanged. (TIF) [file pone.0056381.s003.tif]

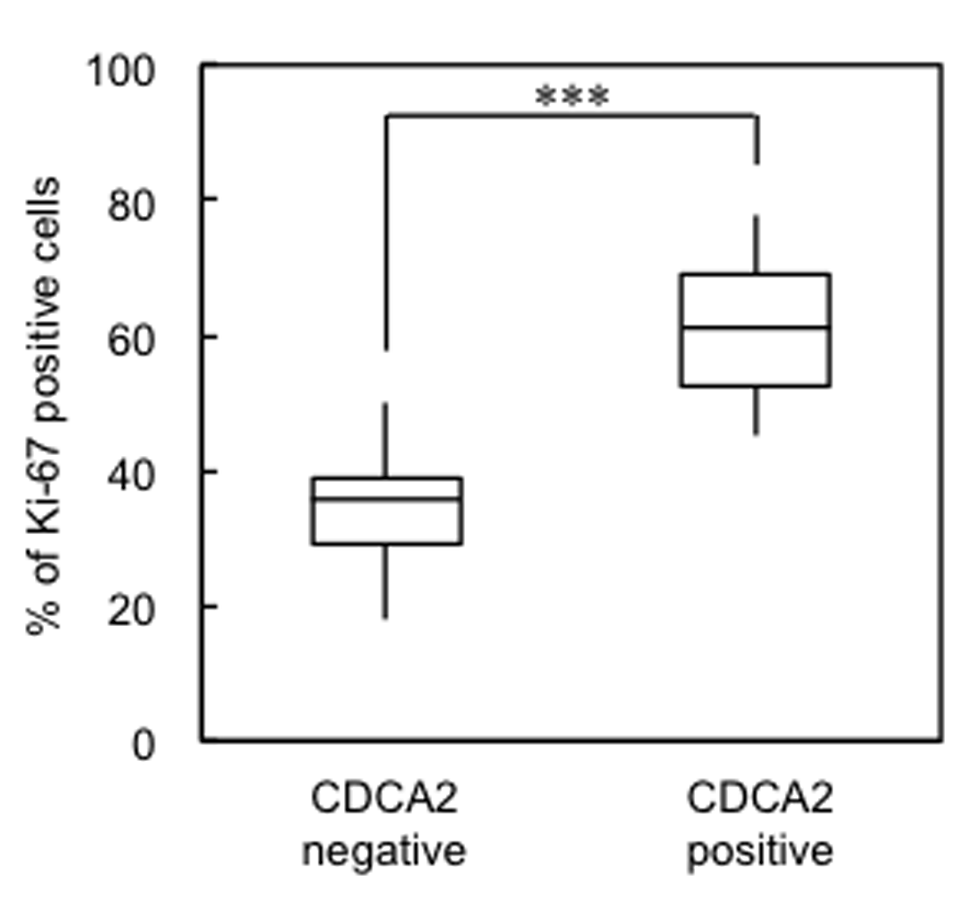

Supplement: Figure S4 — Evaluation of Ki-67 expression in CDCA2 negative and positive group. The percentage of cells stained positive for Ki-67 on 46 OSCCs that were extracted at random from the CDCA2 negative group (23 cases) and CDCA2 positive group (23 cases) based on the IHC. The number of cells stained positive for Ki-67 in CDCA2 positive group significantly higher (***P<0.001, Mann-Whitney's U test) than that in CDCA2 negative group. (TIF) [file pone.0056381.s004.tif]
